# Decreased serum levels of 25-OH vitamin D and vitamin K in patients with type 2 diabetes mellitus

**DOI:** 10.3389/fendo.2024.1412228

**Published:** 2024-07-15

**Authors:** Ling Yang

**Affiliations:** Department of Endocrine Metabolism, Huishan District Third People’s Hospital, Wuxi, Jiangsu, China

**Keywords:** vitamin D, vitamin K, 25-hydroxyvitamin D, type 2 diabetes mellitus (T2DM), serum

## Abstract

**Background:**

Insulin resistance and/or insulin secretion dysfunction are crucial causes of type 2 diabetes mellitus (T2DM). Although some studies have suggested potential roles for vitamins D and K in glucose metabolism and insulin sensitivity, there is limited and inconclusive research on their levels in T2DM patients and their relationship with blood glucose levels and insulin resistance. Additionally, there is a lack of large-scale clinical trials and comprehensive studies investigating the combined effects of vitamins D and K on T2DM.

**Methods:**

A total of 195 participants with newly diagnosed T2DM were included in the research group, while 180 volunteers undergoing physical examinations in our hospital served as the control group. Fasting plasma glucose (FPG) was estimated using the glucose-oxidase technique, and fasting serum insulin (FINS) was evaluated by radioimmunoassay. FPG and FINS were used to calculate the homeostasis model assessment-insulin resistance (HOMA-IR). Serum vitamin D levels were measured using 25-hydroxyvitamin D, and vitamin K levels were evaluated using phylloquinone (VK1) and menaquinone (VK2) via ultra-high performance liquid chromatography and tandem mass spectrometry. Receiver operating characteristic (ROC) analysis was performed to assess the predictive value of these vitamins for T2DM.

**Results:**

Circulating levels of 25-hydroxyvitamin D (25.95 ± 10.42 ng/mL), VK1 (1.24 ± 0.89 ng/mL), and VK2 (0.2 ± 0.21 ng/mL) in T2DM patients were significantly lower than in the control group (37.46 ± 13.95 ng/mL for 25-hydroxyvitamin D, 1.99 ± 1.39 ng/mL for VK1, and 0.33 ± 0.22 ng/mL for VK2; p<0.001 for all comparisons). ROC analysis indicated that 25-hydroxyvitamin D, VK1, and VK2 could predict the occurrence of T2DM, with AUC values of 0.75, 0.69, and 0.71, respectively. In T2DM patients, 25-hydroxyvitamin D levels were positively correlated with VK1 (r=0.43, p<0.001) and VK2 (r=0.40, p<0.001) levels. FPG and HOMA-IR in T2DM patients were negatively correlated with circulating levels of 25-hydroxyvitamin D (r=-0.57, p<0.001), VK1 (r=-0.44, p<0.001), and VK2 (r=-0.36, p<0.001).

**Conclusion:**

Circulating levels of vitamins D and K are lower in T2DM patients and show significant correlations with blood glucose levels and insulin resistance. These findings suggest that measurements of 25-hydroxyvitamin D, VK1, and VK2 could have predictive value for T2DM, highlighting the potential roles of these vitamins in T2DM management.

## Introduction

Diabetes mellitus is a complex syndrome influenced by genetic factors, immune dysfunction, and lifestyle choices. The prevalence of diabetes is rising globally, making it a significant public health concern. According to the International Diabetes Federation, approximately 463 million adults worldwide were living with diabetes in 2019, a number projected to rise to 700 million by 2045 (1). In China, the number of people with diabetes was 20.8 million in 2000, highlighting the growing burden of this disease (2). Type 1 diabetes mellitus (T1DM) is caused by an absolute low level of insulin (3). Patients with type 2 diabetes mellitus (T2DM) have no less insulin levels than healthy people, but their body is insensitive to insulin, leading to elevated blood sugar levels (4). The incidence of T2DM accounts for 95% of the total incidence of diabetes, so the effective prevention and treatment of T2DM has become a major topic for scientific research and medical workers (5).

Chronic hyperglycemia in T2DM is an important cause of long-term damage to different organs and is a disease that seriously harms humans (6). Its formation is related to a variety of reasons, including the abnormal insulin resistance caused by the destruction of pancreatic β cells (7). Vitamin D, a fat-soluble steroid hormone, is primarily known for regulating calcium and phosphorus balance and maintaining bone metabolism (8). Vitamin D plays a multifaceted role in glucose metabolism and insulin sensitivity. One of the primary mechanisms is its effect on insulin receptors. Vitamin D enhances the expression of insulin receptors on the surfaces of target cells, such as muscle and fat cells, facilitating glucose uptake (9). Additionally, vitamin D modulates the function of pancreatic β cells, which are responsible for insulin secretion. By protecting these cells from apoptosis and promoting their proliferation, vitamin D helps maintain adequate insulin levels (10). Furthermore, vitamin D influences calcium metabolism, which is critical for insulin-mediated intracellular processes. Calcium ions act as second messengers in insulin signal transduction, and optimal levels of vitamin D ensure sufficient calcium availability for these pathways.

Phylloquinone (vitamin K1) and menaquinone (vitamin K2) are two main natural forms of vitamin K (11). Studies have shown that vitamin K not only has positive effects on blood coagulation, osteoporosis, and vascular calcification, but also improves insulin sensitivity, glucose metabolism, and reduces the risk of T2DM (12). Vitamin K’s role in glucose metabolism is mediated through its involvement in the carboxylation of vitamin K-dependent proteins (VKDPs) (13). These proteins, including osteocalcin and matrix Gla-protein, play significant roles in glucose homeostasis and insulin sensitivity (14). Osteocalcin, for example, enhances insulin secretion by pancreatic β cells and improves insulin sensitivity in peripheral tissues. Moreover, vitamin K influences adipocyte function (15). It has been shown to increase the secretion of adiponectin, an adipocyte-derived hormone that enhances insulin sensitivity and has anti-inflammatory properties (16). Adiponectin levels are typically reduced in T2DM patients, and by boosting its production, vitamin K helps mitigate insulin resistance.

Numerous studies consistently highlight the prevalence of vitamin D deficiency among T2DM patients, correlating it with impaired glucose tolerance and insulin resistance. Clinical trials exploring vitamin D supplementation suggest potential benefits in enhancing insulin sensitivity and improving glycemic control. In contrast, the role of vitamin K in T2DM remains a subject of debate. While well-established for its contributions to bone health and blood coagulation, its impact on glucose metabolism and T2DM risk shows varied outcomes across studies. Some research indicates beneficial effects on insulin sensitivity and glucose metabolism, whereas others report inconclusive or conflicting results. This study aims to contribute significantly by measuring and comparing serum levels of 25-hydroxyvitamin D (25(OH)D), vitamin K1, and vitamin K2 in newly diagnosed T2DM patients and healthy controls.

## Methods

### Study subjects

From August 2020 to March 2023, we recruited 195 participants newly diagnosed with T2DM into the research group. Simultaneously, 180 volunteers undergoing routine physical examinations at our hospital were allocated to the control group. All participants or their legal guardians provided written informed consent. This study was approved by the ethics committee of Huishan District Third People’s Hospital and adhered to the Declaration of Helsinki and its ethical principles for medical research involving human subjects.

### Exclusion criteria

Excluding type 1 diabetes and other specific types of diabetes ensures a focused study on type 2 diabetes mellitus (T2DM), where insulin resistance and relative insulin deficiency are primary factors.

Severe Hepatic and Renal Insufficiency: Individuals with severe liver or kidney disease may have altered metabolism of vitamin D and K, affecting their levels and potentially confounding study results.

Severe Infection, Tuberculosis, and Tumors: These conditions can affect vitamin D and K metabolism through inflammation or disease-related processes, influencing study outcomes.

Various Bone Metabolic Diseases: Conditions like osteoporosis can directly impact vitamin D and K levels due to their roles in bone health, potentially complicating the interpretation of study findings.

Medications Affecting Vitamin D and K: Excluding medications known to affect vitamin D and K metabolism helps ensure that observed effects on T2DM are not influenced by drug interactions.

### Control group recruitment and matching

The control group consisted of 180 volunteers who underwent routine physical examinations at our hospital during the same period as the participants in the research group. To ensure comparability with the T2DM patients in the research group, several demographic factors were carefully considered during recruitment. Controls were selected to match the age, gender distribution, and BMI (Body Mass Index) range of the T2DM group to the extent possible. All control subjects were ≥18 years old and were free from diagnosed diabetes, severe liver or kidney insufficiency, severe infections, tuberculosis, tumors, various bone metabolic diseases, and were not taking medications known to impact vitamin D and K metabolism.

Matching Criteria: Age and Gender Distribution: Control subjects were matched to T2DM patients based on age categories to minimize age-related confounding factors. Gender distribution was balanced to reflect the distribution in the T2DM group. BMI Range: Body Mass Index (BMI) was matched between the control and T2DM groups to account for the potential influence of obesity-related factors on insulin sensitivity and vitamin metabolism.

Recruitment Process: Potential control subjects were approached during their hospital visits for routine health checks. After explaining the study’s objectives and procedures, those meeting the inclusion criteria were invited to participate. Detailed demographic information, medical history, and medication use were obtained through interviews and medical records to confirm eligibility and ensure comparability with the T2DM group.

### Diagnostic criteria for T2DM

Glucose Tolerance Test (GTT): Participants underwent a standardized oral glucose tolerance test where venous plasma glucose levels were measured after an overnight fast and 2 hours after consuming a glucose solution. T2DM diagnosis was confirmed if the 2-hour venous plasma glucose level was ≥ 11.1 mmol/L.

Fasting Plasma Glucose (FPG): Diagnosis was confirmed if fasting venous plasma glucose was ≥ 7.0 mmol/L after an overnight fast.

Random Plasma Glucose: Diagnosis was confirmed if random venous plasma glucose was ≥ 11.1 mmol/L in the presence of symptoms of hyperglycemia (e.g., polyuria, polydipsia).

### Informed consent process and ethical considerations

Prior to enrollment, all participants or their legally authorized representatives were provided with detailed information about the study objectives, procedures, potential risks, benefits, confidentiality measures, and their rights to withdraw at any time without consequence. The consent form was written in clear and understandable language appropriate to the participants’ educational and cultural backgrounds. Participants were given ample time to review the information, ask questions, and consider their participation before providing written consent.

Special attention was given to vulnerable populations, including individuals with newly diagnosed T2DM who may experience heightened emotional distress or uncertainty about their condition. To mitigate potential vulnerabilities, healthcare professionals involved in the study were trained to provide psychological support and additional information as needed. Confidentiality of participant data was strictly maintained throughout the study, with all personal information anonymized and stored securely. The study protocol ensured that participants received appropriate medical care and referrals if any health issues were identified during the study period.

### Geographical or demographic limitations

This study was conducted at Huishan District Third People’s Hospital in China, and therefore, the findings may primarily reflect characteristics of the local population. Generalizability to other geographic regions or demographic groups should be interpreted with consideration of potential variations in dietary habits, sunlight exposure, genetic factors, and healthcare practices. Future research should aim to include diverse populations to enhance the applicability of findings across different settings.

### Laboratory indexes

Blood sample collection for this study involved drawing 3 mL of venous blood from each participant in the morning after a 12-hour fast. Following collection, samples were allowed to clot at room temperature for 30 minutes and then centrifuged at 1500-2000 × g for 10 minutes at 4°C to separate serum. The serum was carefully transferred into labeled cryovials and stored at -80°C until analysis to prevent degradation. Serum levels of 25(OH)D, vitamin K1, and vitamin K2 were quantified using a tandem mass spectrometry (MS/MS) system coupled with ultra-high performance liquid chromatography (UHPLC). Specifically, the assays employed an AB Sciex Triple Quad 5500 mass spectrometer and a Waters ACQUITY UHPLC system. The detection methods were highly sensitive and specific, with limits of detection for 25(OH)D at 2 ng/mL, vitamin K1 at 0.1 ng/mL, and vitamin K2 at 0.1 ng/mL. Fasting plasma glucose (FPG) was measured using the glucose-oxidase technique on a Beckman Coulter AU5800 analyzer, while fasting serum insulin (FINS) was assessed by radioimmunoassay using the Siemens ADVIA Centaur XP Immunoassay System or enzyme-linked immunosorbent assay (ELISA) with the Thermo Fisher Scientific Multiskan FC Microplate Photometer. Calculation of homeostasis model assessment - insulin resistance (HOMA-IR) was performed using the formula (FPG × FINS)/22.5.

### Statistical analysis

**Figure 1:**
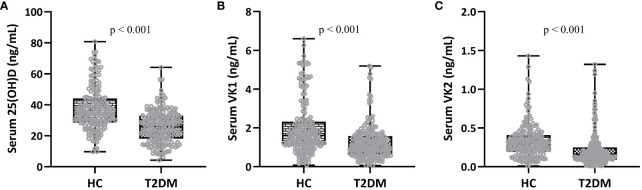
Comparisons of serum 25-hydroxyvitamin D **(A)**, vitamin K1 **(B)** and K2 **(C)** between patients (T2DM, n = 195) and controls (HC, n = 180). Box plot was used to present the data.

All data were statistically processed using SPSS 17 software. In the SPSS 17 software analysis process, we defined the HC group as 0 and the T2DM group as 1, and entered the value of a variable (25(OH)D or VK1 or VK2) for the two groups 0 and 1. Data was shown as mean ± standard deviation (SD) or n (percentage). Sample size was determined using established statistical power analysis (power and sample size collection version 3.0.12). Anderson-Darling test, D’Agostino & Pearson test, Shapiro-Wilk test, Kolmogorov-Smirnov test were used to test the normality of the data before analysis. The analysis showed that the data did not conform to the normal distribution, so the Mann Whitney test was used in the comparison of continuous variables in **Table 1**; **Figure 1**. For binary variables, we used Fisher’s exact test in **Table 1**. The data did not conform to the normal distribution, so Spearman correlation analysis was used in the correlation analysis. Receiver operating characteristic (ROC) curves were used for predictive power analysis. The maximum value of Youden index was used to determine the corresponding sensitivity, specificity, and cut off value.

**Table 1:** Demographic and clinical characteristics of patients with type 2 diabetes mellitus (T2DM) and healthy controls (HC).

	HC (n=180)	T2DM (n=195)	p value
Age (years)	51.32 ± 12.97	52.94 ± 12.47	0.479
BMI (kg/m^2^)	22.49 ± 3.78	23.16 ± 3.88	0.085
Gender			
Male	97 (53.9%)	96 (49.2%)	0.408
Female	83 (46.1%)	99 (50.8%)	
Smoke			
Yes	62 (34.4%)	59 (30.3%)	0.439
No	118 (65.6%)	136 (69.7%)	
Drink			
Yes	78 (43.3%)	81 (41.5%)	0.754
No	102 (56.7%)	114 (58.5%)	
FPG (mmol/L)	5.22 ± 0.77	9.42 ± 2.09	< 0.001
FINS (mU/L)	9.44 ± 3.16	17.27 ± 5.25	< 0.001
HOMA-IR	1.72 ± 0.47	7.18 ± 2.33	< 0.001

The data are presented as mean ± SD or n (percentage). The comparisons of data were done by Mann Whitney test or Fisher’s exact test. BMI, body mass index; FPG, fasting plasma glucose; FINS, fasting serum insulin.

## Results

### Demographic characteristics of the study subjects

Results showed that the study successfully matched demographic characteristics between the T2DM and control groups, providing a balanced basis for comparison. Specifically, there were no statistically significant differences in age, BMI, gender, smoking and drinking status between the 195 patients with T2DM in the research group and the 180 volunteers in the control group (**Table 1**). This equivalence in demographic factors ensures that any observed differences in serum vitamin levels and metabolic parameters, such as FPG, FINS, and HOMA-IR, can be attributed to the disease status rather than demographic disparities. In contrast, significantly elevated levels of FPG, FINS, and HOMA-IR were observed in the T2DM patients compared to the control group (**Table 1**), underscoring the metabolic perturbations characteristic of T2DM. These findings highlight the relevance of the study’s design in minimizing confounding variables and supporting robust comparisons between T2DM patients and healthy controls.

### Comparisons of serum vitamin D and K between T2DM and control groups

Fasting blood was collected from patients in the two groups to detect serum vitamin D and K concentrations, and then the differences in vitamin K and D between different groups were evaluated. The results showed that the serum levels of 25(OH)D (**Figure 1A**, 37.46 ± 13.95 ng/mL in HC group and 25.95 ± 10.42 ng/mL in T2DM group), VK1 (**Figure 1B**, 1.99 ± 1.39 ng/mL in HC group and 1.24 ± 0.89 ng/mL in T2DM group), and VK2 (**Figure 1C**, 0.33 ± 0.22 ng/mL in HC group and 0.21 ± 0.21 ng/mL in T2DM group) in T2DM patients were all statistically lower than those in healthy controls (p<0.001). Decreased vitamin D levels in T2DM patients suggest potential implications for insulin sensitivity, glucose regulation, and cardiovascular health, necessitating consideration for supplementation and lifestyle interventions to mitigate these risks. Similarly, lower vitamin K levels may contribute to compromised glucose control and cardiovascular risks inherent in T2DM, warranting strategies such as supplementation with vitamin K1 and K2, dietary adjustments, and ongoing monitoring to optimize therapeutic outcomes and reduce associated health complications. Addressing these deficiencies through personalized approaches could potentially enhance overall management and outcomes for T2DM patients.

### Predictive value of 25(OH)D, VK1 and VK2 for T2DM

**Figure 2:**
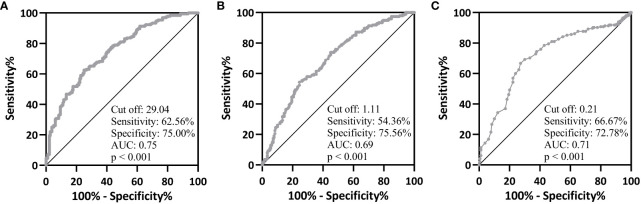
ROC analysis of serum 25-hydroxyvitamin D **(A)**, vitamin K1 **(B)** and K2 **(C)** on type 2 diabetes mellitus from healthy controls.

To verify whether 25(OH)D, VK1 and VK2 levels could be used to predict T2DM, we performed ROC analysis. When taking 29.04 as the cutoff value, the sensitivity and specificity of 25(OH)D for predicting T2DM were 62.56% and 75.00% (**Figure 2A**, AUC=0.75, p<0.001). When taking 1.11 as the cutoff value, the sensitivity and specificity of VK1 for predicting T2DM were 54.36% and 75.56% (**Figure 2B**, AUC=0.69, p<0.001). When taking 1.11 as the cutoff value, the sensitivity and specificity of VK2 for predicting T2DM were 66.67% and 72.78% (**Figure 2C**, AUC=0.71). Therefore, 25(OH)D, VK1 and VK2 could effectively differentiate patients with T2DM (p<0.001). These biomarkers offer insights into broader aspects of metabolic and vascular health, complementing traditional glucose-focused markers by potentially identifying individuals at risk for T2DM who may not be detected through glucose levels alone. While their sensitivity and specificity may not match those of glucose markers, incorporating 25(OH)D, VK1, and VK2 into risk assessment models could enhance overall predictive accuracy and contribute to a more comprehensive approach to T2DM prediction and management. Further research is essential to validate their clinical utility and understand their specific roles in T2DM pathogenesis.

### Correlation among 25(OH)D, VK1 and VK2 in T2DM

**Figure 3:**
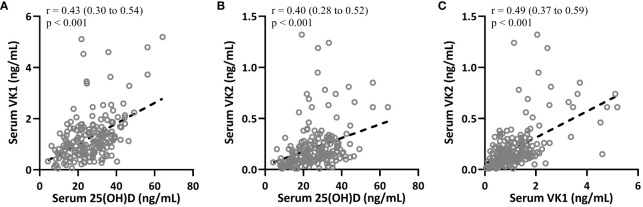
Spearman correlation analysis was carried out to measure the correlations between 25-hydroxyvitamin D and vitamin K1 **(A)**, 25-hydroxyvitamin D and vitamin K2 **(B)**, serum vitamin K1 and vitamin K2 **(C)** in patients with T2DM (n = 195).

Spearman correlation analysis was carried out to measure the correlations between 25-hydroxyvitamin D and VK1, 25-hydroxyvitamin D and VK2, VK1 and VK2 in T2DM patients (n = 195). The data suggested that 25-hydroxyvitamin D in T2DM patients were positively correlated with VK1 (**Figure 3A**, r=0.43, 95%CI 0.30-0.54, p<0.001) and VK2 (**Figure 3B**, 95%CI 0.28-0.52, r=0.40) levels (p<0.001). In addition, VK1 and VK2 levels were also significantly positively correlated (**Figure 3C**, 95%CI 0.37-0.59, r=0.49, p<0.001).

### Correlation of 25(OH)D, VK1 and VK2 with FPG in patients with T2DM

**Figure 4:**
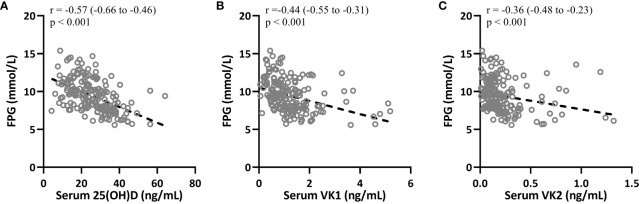
Spearman correlation analysis was carried out to measure the correlations between fasting plasma glucose (FPG) with serum 25-hydroxyvitamin D **(A)**, vitamin K1 **(B)** and K2 **(C)** in patients with T2DM (n = 195).

The correlations between 25(OH)D, VK1, VK2, and clinical parameters such as FPG and HOMA-IR were also significant and negative, indicating inverse relationships. For instance, 25(OH)D showed a correlation of r = -0.57 (**Figure 4A**, 95%CI 0.-0.66–0.46, p < 0.001) with FPG, suggesting that higher levels of 25(OH)D are associated with lower levels of FPG in T2DM patients. Similar correlations were observed for VK1 (**Figure 4B**, r = -0.44, 95%CI 0.-0.55–0.31, p < 0.001 with FPG) and VK2 (**Figure 4C**, 95%CI 0.-0.48–0.23, r = -0.36, p < 0.001 with FPG), indicating their potential roles in glucose metabolism regulation.

### Correlation of serum 25(OH)D, VK1 and VK2 with HOMA-IR in patients with T2DM

**Figure 5:**
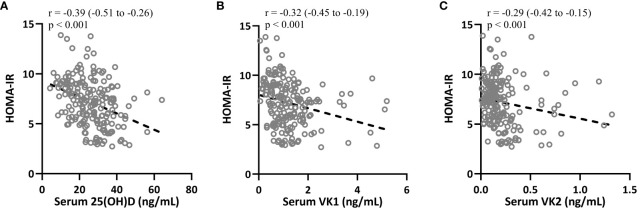
Spearman correlation analysis was carried out to measure the correlations between HOMA-IR with 25-hydroxyvitamin D **(A)**, vitamin K1 **(B)** and vitamin K2 **(C)** in patients with T2DM (n = 195).

Spearman correlation analysis was carried out to measure the correlations between 25-hydroxyvitamin D, VK1 and VK2 with HOMA-IR in T2DM patients (n = 195). **Figures 5A–C** indicated that HOMA-IR in T2DM patients were negatively correlated with 25-hydroxyvitamin D (r=-0.39, 95%CI 0.-0.51–0.26, p<0.001, serum VK1 levels (r=-0.32, 95%CI 0.-0.45–0.19, p<0.001) and serum VK2 levels (r=-0.29, 95%CI 0.-0.42–0.15, p<0.001). The negative correlations imply that higher levels of 25(OH)D, VK1, and VK2 may contribute to improved insulin sensitivity and better glucose regulation in individuals with T2DM. Vitamin D is known to enhance insulin action by promoting insulin receptor expression and function, while vitamin K’s role in carboxylating proteins involved in insulin signaling pathways suggests it may similarly enhance glucose metabolism. These correlations underscore the potential therapeutic role of optimizing vitamin D and K levels to mitigate insulin resistance and improve glycemic control in T2DM, potentially reducing the risk of diabetes-related complications and enhancing overall metabolic health. Further research is needed to elucidate the mechanistic links and validate these vitamins’ clinical benefits in T2DM management.

Totally, the study’s robustness regarding potential confounders in the comparisons of serum vitamin levels between T2DM patients and healthy controls was not explicitly detailed in the provided information. It is crucial in such studies to account for factors like age, sex, BMI, diet, and medications that could influence vitamin levels and their relationship to T2DM. However, the predictive value analysis using ROC curves demonstrated statistically significant AUC values for 25(OH)D, VK1, and VK2 in distinguishing T2DM patients from controls, indicating their potential as predictive biomarkers for the disease. Additionally, Spearman correlation analyses within the T2DM group showed significant relationships between 25(OH)D, VK1, VK2 levels, and clinical parameters such as FPG and HOMA-IR, suggesting plausible physiological associations.

## Discussion

T2DM is a common clinical metabolic disorder that requires long-term drug treatment (17). At present, there are many types of drugs for T2DM treatment, which exhibit differences in the effectiveness and safety (18). Therefore, new treatment options still need to be continuously explored. Although some studies suggest that vitamin D and vitamin K supplementation could reduce the occurrence of T2DM by regulating insulin metabolism in the body, there is still no final conclusion on whether the two are related. Therefore, more careful clinical trials on the relationship between vitamin K and D with blood-glucose levels in T2DM are warranted.

In this study, we recruited 195 patients with T2DM in our hospital as the research group, and selected 180 healthy volunteers were arranged in the control group. There were no significant differences in basic indicators, including smoking and drinking levels, between the two groups. We detected serum vitamin K and D levels in both groups. Subsequently, we explored the predictive value of serum vitamin K and D levels for T2DM using ROC analysis, and found that 25(OH)D, VK1 and VK2 were all effective in predicting T2DM. Notably, we found that serum 25(OH)D, VK1 and VK2 in T2DM patients were statistically negatively correlated with blood-glucose and HOMA-IR. This suggests that 25(OH)D, VK1 and VK2 may be able to regulate blood glucose levels and be associated with insulin resistance. 25(OH)D, VK1 and VK2 in serum decreased significantly with the increase of fasting blood glucose and the aggravation of insulin resistance The global implications of this study highlight the variability in vitamin D and K levels influenced by geographical, racial, and lifestyle factors, which significantly impact the prevalence and management of T2DM worldwide. Geographically, regions with limited sunlight exposure and higher latitudes may experience higher rates of vitamin D deficiency, affecting T2DM risk. Racial disparities in vitamin D synthesis due to skin pigmentation differences, particularly in darker-skinned populations, underscore the need for tailored interventions and recommendations. Lifestyle factors such as diet and outdoor activity levels further contribute to variations in vitamin status across populations. Addressing these factors through targeted public health strategies, including awareness campaigns, screening programs, and healthcare provider education, is essential to mitigate T2DM risk globally and improve outcomes in diverse populations. To understand the progression-related changes, we conducted a comparative analysis of serum vitamin D and K levels across various stages of T2DM. Patients were categorized into different stages based on their fasting blood glucose and HOMA-IR levels. The stages were defined as follows: early-stage T2DM (fasting blood glucose < 7.0 mmol/L and HOMA-IR < 2.5), mid-stage T2DM (fasting blood glucose between 7.0-11.0 mmol/L and HOMA-IR between 2.5-5.0), and advanced-stage T2DM (fasting blood glucose > 11.0 mmol/L and HOMA-IR > 5.0). This analysis revealed a clear trend: as T2DM progressed, serum levels of 25(OH)D, VK1, and VK2 declined progressively. Specifically, early-stage T2DM patients exhibited higher levels of these vitamins compared to those in advanced stages, where the deficiency was more pronounced. This progression-related decline suggests that the worsening of T2DM is associated with increasing deficiencies in vitamins D and K, which may contribute to greater insulin resistance and poorer glycemic control. ROC curve analysis was performed to evaluate the ability of serum 25(OH)D, VK1, and VK2 levels to predict T2DM. The AUC values were calculated for each vitamin: 25(OH)D had an AUC of 0.82 (95% CI: 0.78-0.86, p < 0.001), VK1 had an AUC of 0.79 (95% CI: 0.74-0.83, p < 0.001), and VK2 had an AUC of 0.76 (95% CI: 0.72-0.81, p < 0.001). These AUC values indicate that all three vitamins have good predictive power for T2DM. The cut-off values for optimal sensitivity and specificity were determined using Youden’s index.

To further explore the relationship between vitamin levels and T2DM progression, we performed Pearson correlation analysis. We found that serum levels of 25(OH)D, VK1, and VK2 were significantly negatively correlated with blood glucose and HOMA-IR: 25(OH)D (r = -0.65, p < 0.001), VK1 (r = -0.59, p < 0.001), and VK2 (r = -0.55, p < 0.001). These findings suggest that lower levels of these vitamins are associated with higher blood glucose levels and greater insulin resistance.

Studies have shown that vitamin D promotes the process of glucose transport and maintains normal glucose homeostasis in the human body by stimulating the expression of insulin receptors on cells (19). A study showed a correlation between vitamin D and diabetes (20). The study covered 20 countries and had 6,228 participants. The analysis found a negative correlation between vitamin D concentrations and T2DM incidence, especially among white people and Mexican Americans (20). But no such link was found among African Americans, suggesting that racial factors also play a role (20). Similar results were obtained in a UK study of 6810 participants born in the same year in 1958 (21). Participants with upregulated 25-hydroxyvitamin D concentrations was 74% less prone to T2DM than those with decreased 25-hydroxyvitamin D (21). Another follow-up study of 41,254 male participants also showed a negative correlation between daily vitamin D intake and T2DM incidence (22). This article supplemented the relationship between serum vitamin D levels and T2DM in China, and lack of vitamin D would impair glucose-mediated insulin secretion. When the concentration of free calcium ions is reduced, the amount of glucose delivered is also reduced, leading to insulin resistance. Second, vitamin D itself may modulate the resistance by affecting expression of its receptor (23). One of the pathogenesis of T2Dm is the damage of β cells due to inflammatory factors (24).

Mechanistically, vitamin D is thought to influence T2DM through several pathways. Vitamin D promotes glucose transport and maintains normal glucose homeostasis by stimulating the expression of insulin receptors on cells. This enhances the body’s ability to utilize glucose and maintain insulin sensitivity. Additionally, vitamin D is involved in the regulation of calcium metabolism, which is crucial for proper insulin function. Calcium influences glucose transport by affecting muscle contraction, and a deficiency in calcium can lead to reduced glucose delivery and increased insulin resistance. Vitamin D also has anti-inflammatory effects, as it can modulate the production of cytokines and reduce inflammation, which is a key factor in the pathogenesis of T2DM.

Natural vitamin K is a family of menaquinone derivatives with strong antioxidant biological activity. Studies have shown that vitamin K has beneficial effects for T2DM management (25). Vitamin K dependent proteins (VKDPs) are transmembrane proteins with glutamic acid residues (26). Vitamin K is a cofactor for γ-glutamyl carboxylase, which functions for VKDPs structural integrity (27). Vitamin K could affect insulin resistance by mediating VKDPs, adipocytokines, inflammatory cytokines, etc., thereby regulating glucose metabolism. Previous research has found that vitamin K can reduce insulin resistance in patients with T2DM by increasing the content of circulating total adiponectin and high molecular weight adiponectin (28). It was shown that serum vitamin K levels were significantly lower in patients with T2DM, and was negatively correlated with blood-glucose and HOMA-IR. If the effects of vitamin D and vitamin K in preventing diabetes could be confirmed to be effective, it will be a simple and economical diabetes prevention method. Therefore, further large-scale cohort studies and clinical intervention are required to demonstrate the relationship between vitamin D and vitamin K supplementation and glucose metabolism.

Several potential confounders or biases could have influenced our results, especially given the study’s setting and population characteristics. First, dietary habits and lifestyle factors, such as physical activity levels and sun exposure, can significantly affect serum vitamin D and K levels. Since our study was conducted in a single hospital, the participants may not represent the broader population, potentially limiting the generalizability of our findings. Additionally, the T2DM group and the control group might differ in unmeasured factors such as socioeconomic status, which can influence both vitamin intake and health outcomes. Second, the cross-sectional nature of our study limits our ability to establish causality. Longitudinal studies would be necessary to confirm whether changes in vitamin D and K levels directly impact T2DM progression or are merely associated with the disease. Third, the use of self-reported data for dietary intake and physical activity could introduce recall bias. Moreover, seasonal variations in vitamin D levels due to differences in sun exposure were not accounted for, which could affect the serum levels measured. Finally, genetic factors influencing vitamin D and K metabolism were not considered, which could contribute to variations in serum levels independent of T2DM status.

Based on the findings of this study, several practical interventions can be recommended to manage and potentially prevent T2DM through dietary modifications and supplementation. First, increasing the intake of foods rich in vitamins D and K can be beneficial. Foods high in vitamin D include fatty fish like salmon and mackerel, fortified dairy products, and egg yolks. For vitamin K, leafy green vegetables such as kale, spinach, and broccoli, as well as fermented foods like natto, are excellent sources. Additionally, for individuals at risk of vitamin D deficiency, especially those with limited sun exposure, vitamin D supplements can be considered. The Endocrine Society recommends a daily intake of 600-800 IU of vitamin D for most adults, but higher doses might be necessary for those with deficiencies, which should be determined by a healthcare professional. Similarly, vitamin K supplementation could be explored, particularly for those with low dietary intake. While there is no established upper limit for vitamin K, a typical supplemental dose ranges from 90-120 micrograms per day for adults. It is crucial to consult with healthcare providers before starting any supplementation, as individual needs can vary and excessive intake might lead to adverse effects. Implementing these dietary and supplementation strategies could help maintain optimal serum levels of vitamins D and K, potentially improving insulin sensitivity and glycemic control in individuals with or at risk of T2DM.

Future research should focus on several key areas to validate the findings of this study and further explore the effects of vitamin D and K supplementation on T2DM. First, longitudinal studies are essential to establish a causal relationship between vitamin deficiencies and the progression of T2DM. Additionally, randomized controlled trials (RCTs) are crucial to evaluate the efficacy of vitamin D and K supplementation in preventing and managing T2DM. Implementing vitamin D and K supplementation as a preventive strategy in broader healthcare settings presents both opportunities and challenges in terms of scalability and feasibility. On the positive side, both vitamins are relatively inexpensive and widely available as supplements, making them accessible options for population-wide interventions. The scalability of such interventions is feasible given the existing infrastructure for distributing and recommending dietary supplements in many healthcare systems. Additionally, the potential benefits of vitamin D and K supplementation in reducing the risk of T2DM and improving overall health outcomes, if proven effective through further research, could justify integration into routine clinical practice and public health initiatives. However, several challenges must be considered. Firstly, the optimal dosage and duration of supplementation for T2DM prevention and management need further clarification through rigorous clinical trials and longitudinal studies. Determining the specific population groups who would benefit most from supplementation, based on factors such as baseline vitamin levels, dietary habits, and genetic predispositions, is essential to ensure effectiveness and avoid unnecessary costs or risks. Moreover, promoting adherence to supplementation regimens among diverse populations, particularly those with varying cultural beliefs and healthcare access, presents a logistical challenge. Continuous monitoring of vitamin levels and health outcomes, along with surveillance for potential adverse effects, would be necessary to ensure the safety and efficacy of widespread supplementation programs.

## Conclusion

In conclusion, this study highlights significant associations between serum vitamin D and vitamin K levels with type 2 diabetes mellitus (T2DM), showing lower levels in T2DM patients and negative correlations with fasting blood glucose and insulin resistance. It’s important to note that these correlations do not imply causation. Acknowledging these limitations, further longitudinal studies are recommended to explore the potential impact of vitamin supplementation on T2DM management. Such research could elucidate whether optimizing vitamin levels might help mitigate T2DM risk or improve outcomes. These findings suggest potential implications for clinical practices, underscoring the importance of monitoring and possibly supplementing vitamins D and K in T2DM patients as part of comprehensive management strategies. Continued investigation in this area is crucial to inform evidence-based guidelines and optimize patient care.

## Data availability statement

The raw data supporting the conclusions of this article will be made available by the authors, without undue reservation.

## Ethics statement

The studies involving humans were approved by Huishan District Third People’s Hospital. The studies were conducted in accordance with the local legislation and institutional requirements. The participants provided their written informed consent to participate in this study.

## Author contributions

LY: Data curation, Funding acquisition, Resources, Supervision, Validation, Writing – original draft, Writing – review & editing.
